# Application of an open-chamber multi-channel microfluidic device to test chemotherapy drugs

**DOI:** 10.1038/s41598-020-77324-3

**Published:** 2020-11-23

**Authors:** Hui-Sung Moon, Chang Eun Yoo, Sangmin Kim, Jeong Eon Lee, Woong-Yang Park

**Affiliations:** 1 Samsung Genome Institute, Samsung Medical Center, Seoul, 06351 South Korea; 2 Department of Breast Cancer Center, Samsung Medical Center, Seoul, 06351 South Korea; 3 Department of Surgery, Samsung Medical Center, Sungkyunkwan University School of Medicine, Seoul, 06351 South Korea; 4 Department of Health Sciences and Technology, Samsung Advanced Institute for Health Sciences & Technology, Sungkyunkwan University, Seoul, 06351 South Korea; 5 Department of Molecular Cell Biology, Sungkyunkwan University School of Medicine, Suwon, 16419 South Korea

**Keywords:** Drug screening, Lab-on-a-chip

## Abstract

The use of precision medicine for chemotherapy requires the individualization of the therapeutic regimen for each patient. This approach improves treatment efficacy and reduces the probability of administering ineffective drugs. To ensure accurate decision-making in a timely manner, anticancer drug efficacy tests must be performed within a short timeframe using a small number of cancer cells. These requirements can be satisfied via microfluidics-based drug screening platforms, which are composed of complex fluidic channels and closed systems. Owing to their complexity, skilled manipulation is required. In this study, we developed a microfluidic platform, to accurately perform multiple drug efficacy tests using a small number of cells, which can be conducted via simple manipulation. As it is a small, open-chamber system, a minimal number of cells could be loaded through simple pipetting. Furthermore, the extracellular matrix gel inside the chamber provides an in vivo-like environment that enables the localized delivery of the drugs to spontaneously diffuse from the channels underneath the chamber without a pump, thereby efficiently and robustly testing the efficacy and resistance of multiple drugs. We demonstrated that this platform enabled the rapid and facile testing of multiple drugs using a small number of cells (~ 10,000) over a short period of time (~ 2 days). These results provide the possibility of using this powerful platform for selecting therapeutic medication, developing new drugs, and delivering personalized medicine to patients.

## Introduction

Cancer is a lethal disease that affects millions of people worldwide and accounts for approximately 13% of all deaths globally^1^. Various factors such as type, grade, and size, are considered during the selection of appropriate therapy, and chemotherapy is often selected for the treatment of many cancers^2^. Although these drugs are clinically approved, and substantial evidence exists to support these standardized regimens^3^, the positive response of an individual is not guaranteed and the response rates to treatment remain insufficient^4,5^ owing to the genetic and environmental diversity of individual patients. Therefore, the development of individualized chemotherapy is imperative to achieve effective treatments^6^.

To increase the effectiveness of treatment, it is necessary to determine the efficacy of selected drugs in a particular patient as quickly as possible to construct or switch chemotherapeutic strategies and enable the timely management of cancer therapy^7^. As a result, there is a great need to develop rapid screening techniques that evaluate the efficacy of drugs, which will aid in the timely stratification of patients as responders or non-responders^8^.

The major hurdle in evaluating drug efficacy for treating tumors from a primary cancer is the low sample availability. Except for some extraordinary cases such as leukemia, the total number of cancer cells acquired from general, small, solid tissue after dissociation may be less than 1 million. To overcome this hurdle, various tumor amplification methods such as spheroid cultures, have been tested, which has increased the success rate for selecting more effective drugs^9–11^. However, there are fundamental concerns regarding amplified tumors—including preserving the genetic uniformity of the original tumors—although aggressive driver gene mutations are preserved in the process of tumor amplification^12^. Therefore, the development of screening techniques that can test a small number of cancer cells without amplification is desirable.

Microfluidics is a promising technology that may help overcome the obstacle of low sample volume input^8,13–15^. As a miniaturization technology with internal dimensions ranging from micrometers to millimeters, a microfluidic platform for drug analysis constitute a miniaturized, *in-vivo*-like analytical environment connected to a 3-dimensional (3-D) cell model cultured on organ microchips^16^. Moreover, it could concurrently provide analytical efficiency and high-throughput screening with minimal consumption of the sample or reagents^17^. Owing to these innovations, the microfluidic technology has the ability to analyze single cells, enabling the drug response to be observed in individual cells^18–20^. Cell-based analysis systems can be miniaturized to examine various properties such as drug resistance and cell–cell communication, owing to their ability to accommodate and control small samples and operate multiplex assays. These cell-based analysis systems can modified into high-throughput microfluidic platforms with various channel network designs^21,22^ or droplet-based fluidics^23,24^. Compared with conventional chamber- and dish-based systems, microfluidic systems can control well-defined conditions and create more realistic in vivo environments via the incorporation of extracellular matrix (ECM) gels, resulting in cells with more relevant morphology, gene/protein expression, and drug reactions^25–27^. Several research groups have also employed spatial and temporal variations to the structure of their microfluidic system^28–30^ to better stimulate and observe complex biological systems that enable cells to be preserved with their in vivo-like phenotypes, resulting in accurate drug responses^31^. Although many technological developments have been made, fully incorporating these developments into the drug-testing microfluidic platform requires complex chip design and detailed manipulation^17^ . Therefore, it is necessary to develop a drug-testing platform that can quickly confirm the effectiveness of a drug using simple operating process that consumes a small amount of each sample.

**Figure 1:**
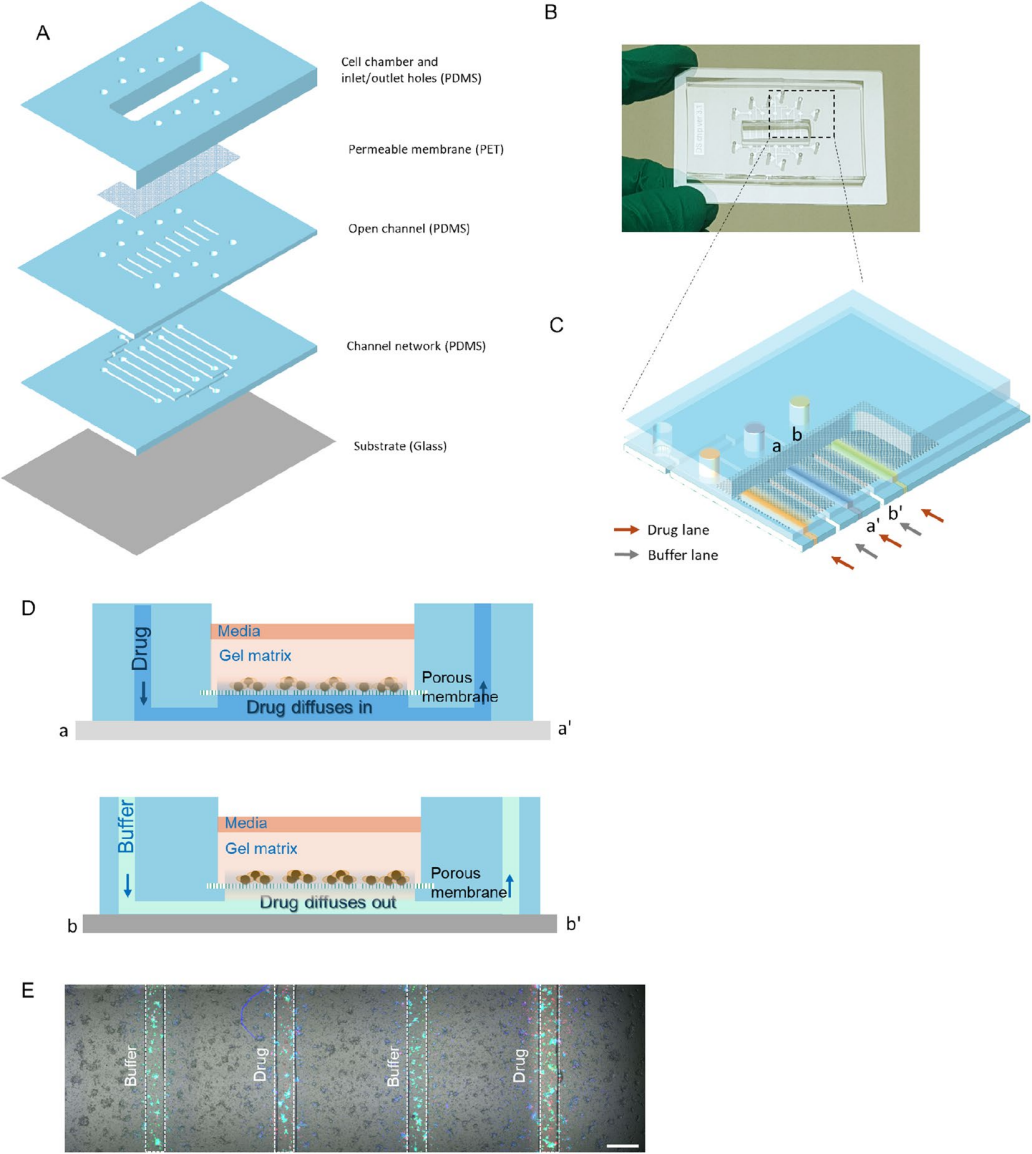
Schematic of the device. (**A**) Layer-by-layer illustration. (**B**) Photograph of the fabricated device. (**C**) Expanded cross-layer view of the working area. Micro-channels filled with color represent the drug-release channels, whereas the transparent channels located between each drug-release channel represent the buffer channels. (**D**) Cross-sectional view and working principal. (**E**) Working image of the device (Scale bar = 50 μm). Dashed lines indicate the location of the drug-release and medium channels underneath the membrane.

In this study, we developed a microfluidic drug-testing platform and established its associated cell manipulation methods to accurately perform multiple drug efficacy tests using a small number of cells, which was conducted by simply pipetting. The platform was designed to have an open chamber and a porous membrane with a microchannel underneath (Fig. 1) to allow conventional pipetting and minimize the use of fluidic wares, thereby providing operational compatibility and convenience^32^. Cells were attached to the membrane and covered with Matrigel to provide an in vivo-like environment and control chemo-invasion in conjunction with the microchannel network. A buffer channel between the drug-release channels enabled the localized delivery of the tested drugs in a cross-contamination-free manner. To confirm the performance of this platform, we conducted a drug efficacy test on approximately 10,000 MCF-7 cells, a breast cancer cell line that has adherent properties. Four total drugs were selected from the AC-T regimen for breast cancer (doxorubicin, paclitaxel, and cyclophosphamide) as well as a commonly used drug in microfluidic cell-based experiments, cisplatin. These drugs were investigated to determine whether the ineffectiveness of anticancer drugs could be detected by our platform. We also performed these tests for another model cancer cell line, K-562, a leukemia cell line with non-adherent properties. This cell number is achievable for rare materials, and the number of drugs tested was adequate to allow the selection of the best chemotherapy regimen among the possible treatment regimens^3^. Finally, we also demonstrated that real-time kinetics for the drug-sensitivity of cancer cells could be determined by monitoring cell viability at the single cell level.

## Results

### Design of the microfluidic device for the chamber-based drug test

In this study, we constructed a PDMS-based microfluidic platform to conduct cell-based drug tests. This platform allowed the localized delivery of drugs to a matrix gel-laden cell chamber at a designated location. As shown in Fig. 1A, the platform comprised five layers, including a chamber, a track-etched membrane, and micro-channels. At the top, in the cell chamber (24 × 6.99 mm), the inlet/outlet holes were bored, and a permeable membrane was bonded between the cell chamber and open channel layer. The open channel layer connected the drug-release channel and buffer channels (width × length × height, F × 5000 μm × 100 μm) to a permeable membrane and the inlet/outlet holes to the channel network. The channel network layer supplied drugs to five drug-release channels via five drug inlets and distributed the medium to four buffer channels, which were introduced from one inlet. At the bottom, a 75 × 50 mm slide glass was bound to the channel network layer as a substrate.

The structure of the fabricated device is shown in Fig. 1B. As the glass substrate is the same size as that of two ordinary glass slides combined and has an open chamber enabling conventional pipetting, our platform offers ease of handling and compatibility with conventional biological experiments. The 3-D cross-sections of the device are illustrated in Fig. 1C. Underneath the membrane, the drug-release and buffer channels were placed alternately. In the device, the drugs diffused from the drug-release channel through the membrane to the bottom of the gel matrix where the cells were located (Fig. 1D). The cell culture medium diffused from the buffer channel, diluted the drugs, and functioned as a concentration sink to achieve localized diffusion. As a result, after drug-release and Live/Dead staining, we obtained segmented reaction regions for the cells, such as the discrete lanes shown in Fig. 1E. In each drug-release channel (i.e., a lane), the cells were exposed to certain drugs. Thus, the efficacy of the drugs was determined by staining to measure cell viability.

### On-chip cell cultivation and manipulation

To operate the device, we loaded a cell suspension with medium on the surface of the membrane in the chamber. The loaded cells (MCF-7 cells) were attached to the membrane and a 2-D layer was first formed during incubation. After 3 h, once the cells were confirmed to adhere to the membrane; the Matrigel was loaded into the chamber to cover the cell layer and fill approximately half of the chamber. As a result, the cells were trapped in the initial position, and the ECM gel provided an in vivo-like environment and a diffusion barrier, which eliminated convection inside the gel. Without the matrix gel, the cells were found to retain their 2-D structure. By staining the cytoplasm, each individual cell could be distinguished (Fig. S1A). Meanwhile, cells within the matrix gel formed a clustered 3-D structure as the incubation proceeded (Fig. S1B).

### Numerical analysis and verification of localized chemo-invasion in the cell chamber

**Figure 2:**
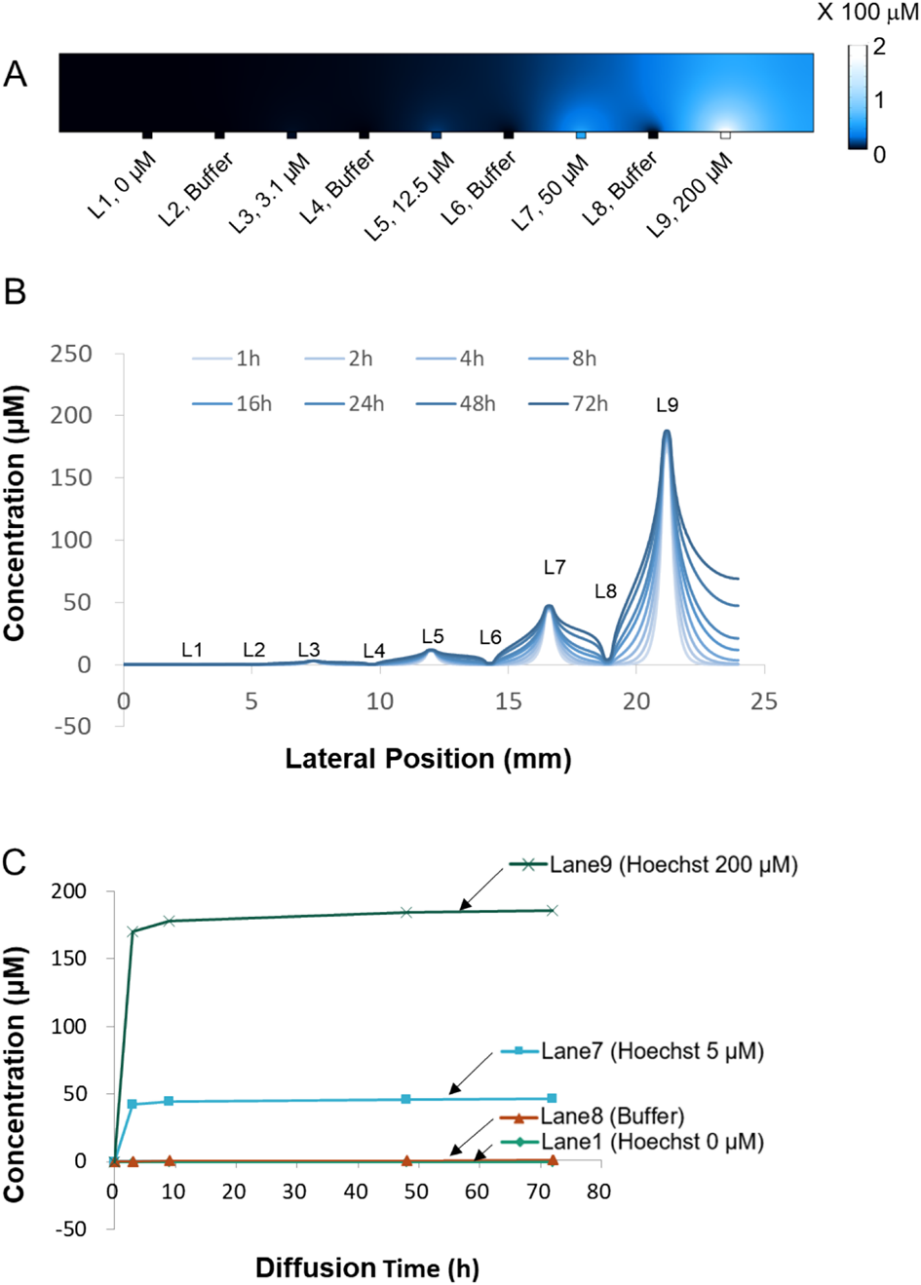
Concentration profile modeled using COMSOL Multiphysics during the localized chemo-invasion of Hoechst from the underlying channel. (**A**) Surface plot of the concentration profile across the device at 48 h. (**B**) Time-dependent concentration profile across the device. (**C**) Concentration change of the region over the sink lane (lane 8) and test lanes (lanes 1, 7, and 9) over time.

As our platform was designed to perform multiplexed testing in a single chamber via localized chemo-invasion in a cross-contamination-free manner, the diffusion profile in the chamber was tested through numerical analysis to confirm our design. With the help of COMSOL Multiphysics, a model was constructed using our device dimensions and material properties. In each drug-release channel (i.e., lanes 1, 3, 5, 7, and 9), 0, 3.1, 12.5, 50, and 200 μM Hoechst 33,342 solution, respectively, was introduced as a boundary condition owing to its similar molecular weight (615.99) to common anticancer drugs. In addition, Hoechst 33,342 can be verified experimentally. As shown in the cross-sectional concentration profile in Fig. 2A, the chemical agent was selectively delivered to the region that overlapped with the drug-release channels. In addition, the buffer channel successively reduced lateral diffusion. The time-dependent concentration profile across the device, 30 μm above the membrane, is shown in Fig. 2B. This result indicates that the concentration at each lane position, i.e., the region of interest, remained stable over time. Further, variations only occurred in the area between the lanes that existed outside the experimental area. The concentration profile of the right region next to lane 9 showed elevated values owing to the absence of a buffer channel, unlike that in the left region adjacent to lane 9. These findings suggest that the buffer channels act as a sink to limit lateral diffusion. As shown in Fig. 2C, the buffer lane between the channels with the highest chemical concentrations (50 μM and 200 μM) expressed the same level of intensity as the control lane (0 μM).

**Figure 3:**
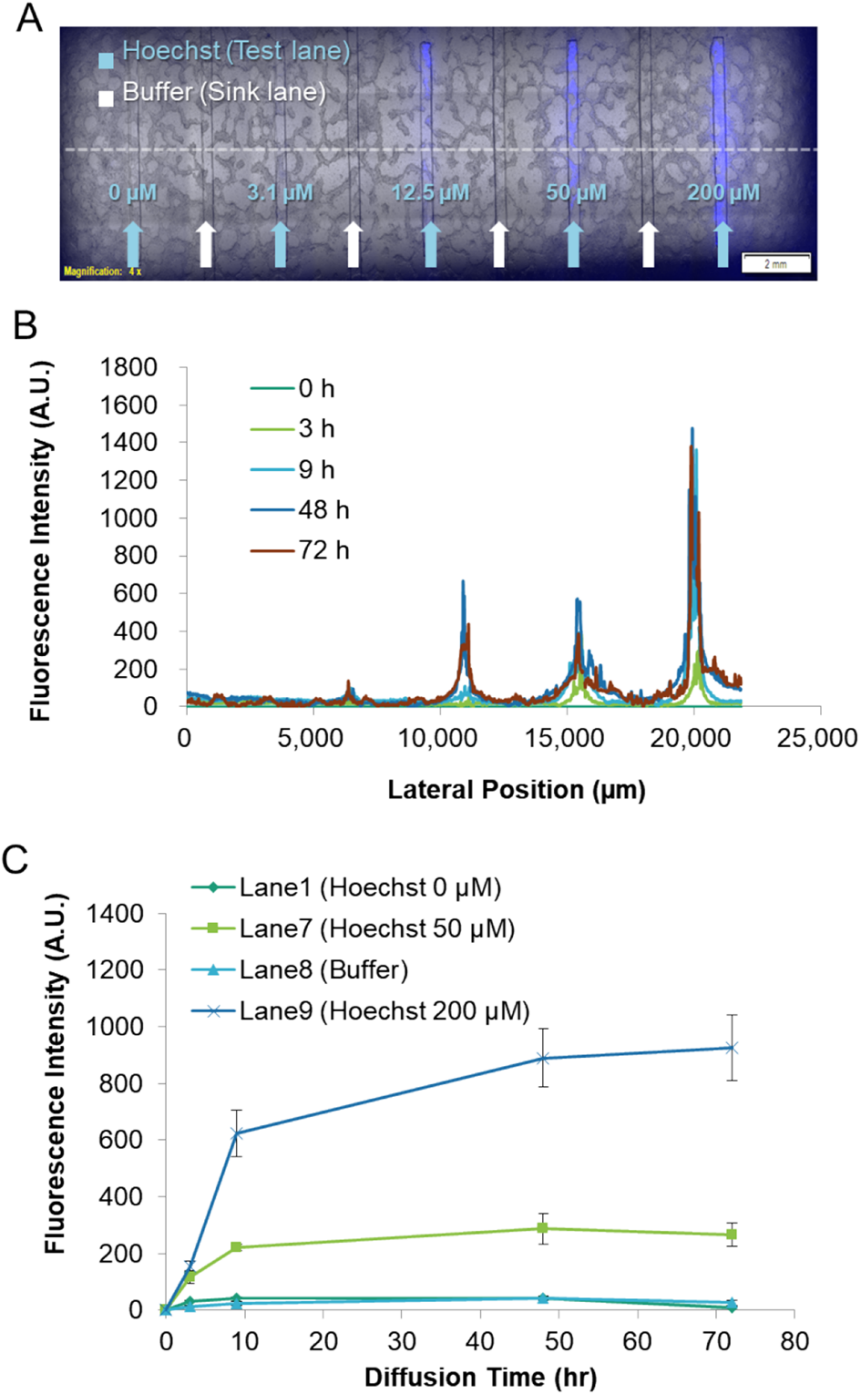
Localized chemo-invasion from the underlying channel into the cells on the porous membrane without cross-contamination. (**A**) Micrograph of cells on the membrane exposed to different doses of Hoechst solution through the test lanes separated by the sink lanes for 72 h. (**B**) 2-D fluorescence intensity profile across the membrane (dashed line in panel (**A**)) at the indicated time intervals. (**C**) Time-dependent fluorescence intensity profile of cells located over the sink lane (lane 8) and test lanes (lanes 1, 7, and 9). The error bars represent the standard deviation for each time point (Average number of cells (n) at each time point is 30).

To verify the localized delivery estimated from the simulation, we visualized the diffusion patterns in the chamber using fluorescence microscopy. First, approximately 10^6^ of MCF-7 cells was loaded to cover the entire area of the chamber surface while Matrigel was loaded to cover the cell area. Thereafter, Hoechst was delivered under the same conditions employed during numerical analysis; this dye was allowed to diffuse within the cell chamber while the medium continued to flow in the buffer channel at 40 μL/h. To measure the diffusion over time, we performed time-lapse fluorescence image acquisition over 72 h. As shown in Fig. 3A, Hoechst was selectively delivered to the region with the drug-release channels beneath it. In the fluorescence intensity profile in Fig. 3A, the intensity peaks depict the location of the drug-release lanes, and the amplitudes are proportional to the concentration of Hoechst staining (Fig. 3B). The fluorescence intensity around the buffer channel was equivalent to the background level, indicating that lateral diffusion was limited. As shown in Fig. 3C, the buffer lane between the highest Hoechst concentration (50 μM, 200 μM) lanes exhibited the same level of intensity as the control lane (0 μM; Fig. 2B). A higher fluorescence concentration indicated a higher fluorescence intensity, and the intensity increased over time. After 48 h, the intensity slightly decreased owing to the degradation of fluorescence. As these experimental results showed adequate agreement with the simulation results, the working concept of our device was validated.

### On-chip drug efficacy test

**Figure 4:**
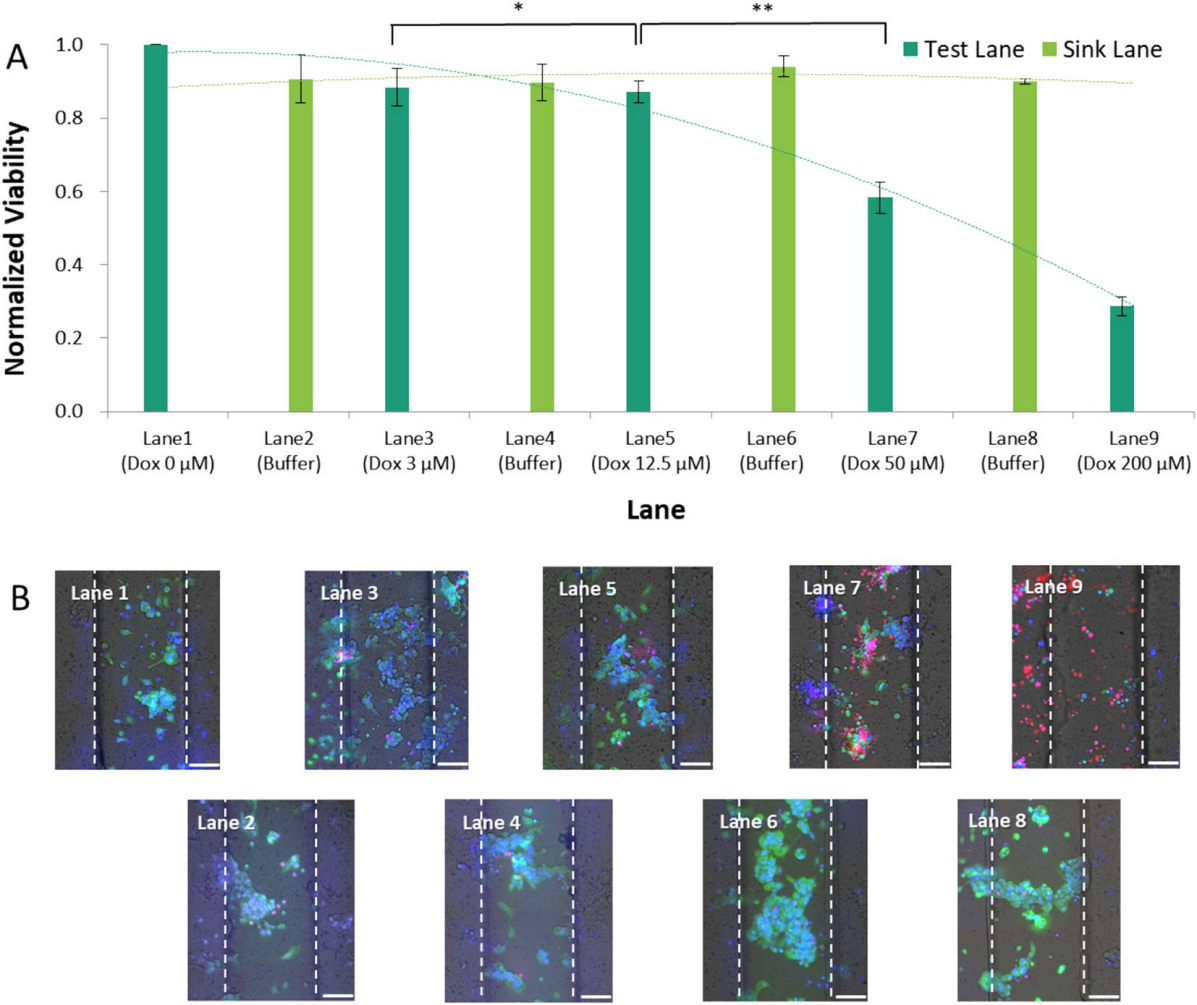
Dose-dependent viability of cells on the membrane. (**A**) Normalized viability of cells in each test lane (lanes 1, 3, 5, 7, and 9) and buffer lane (lanes 2, 4, 6, and 8). The error bars represent the standard deviations for each drug (n = 4). * p-value = 0.819, ** p-value = 0.016. (**B**) Stained image of cells located over the test (odd numbered) and sink (even numbered) lanes. (Blue: Hoechst, Green: calcein-AM, Red: ethidium homodimer I). Dashed lines in each image represent virtual boundaries that indicate lanes loaded with drug (test lane) or buffer (sink lane) (Scale bar = 100 μm).

After verifying the diffusion of the reagent into a restricted region in the cell chamber, we performed a cell viability test following the delivery of the anticancer drug, doxorubicin, to the MCF-7 cells in the chamber of our platform. The viability of MCF-7 cells was lower when a higher concentration of doxorubicin was delivered to the lane (Fig. 4A). However, in the buffer lanes, cell viability remained the same despite the location (p-value of one-way ANOVA between all buffer lanes > 0.05). This finding indicates that there was no effect in the lanes adjacent to the drug-release, even in lane 8, where the drug concentration in the adjacent lane was the highest. By comparing the calculated statistical differences (i.e., p-values) between the viable cells in lanes 3 (3 μM) and 4 (12.5 μM) with cell viability between lanes 4 (12.5 μM) and 5 (50 μM), the viability of the MCF-7 cells began to decrease with 12–50 μM doxorubicin. Further, cell viability was as low as 28.7% as the concentration of doxorubicin increased to 200 μM. Using our platform, the IC_50_ of MCF-7 over doxorubicin was comparable to the value obtained in a recent study using a 3-D context^33^. The Live/Dead stained images in Fig. 4B show the response of the cells to the concentration of the drug. The cells in the control and buffer lanes had a high viability with a clustered configuration, whereas those in the drug-release lanes had a lower viability and greater morphological deterioration as the drug concentration increased. Based on the above results from using our microfluidic platform, we concluded that the efficacy of the drug could be identified in a concentration-dependent manner. Further, cross-contamination due to the lateral diffusion of the drug could be avoided in our device. Additionally, our platform revealed images of morphological changes in single cells that occurred in response to the drug (Fig. 4B).

The number of cells used in this experiment was approximately 10,000. As the device had nine lanes, the number of cells per lane was approximately 1,000 fewer than that in conventional plate-based platforms; however, this number was similar to that used in other microfluidic platforms^2^. The number of cells required for the test may be further reduced by modifying the cell chamber design to include additional compartments.

### On-chip drug efficacy test for different drugs and cell lines

After confirming that changes in cell viability were concentration-dependent of a single drug, which occurred independently and without interference from another concentration, we performed drug screening experiments using MCF-7 cells. To demonstrate the screening performance, we compared four anticancer drugs which have efficacy or ineffectiveness. Doxorubicin (Adriamycin) is widely used drug for cancers and cancer cell lines. On the other hand, paclitaxel (Taxol) is effective for many types of breast cancer cell, however, it is ineffective for cancer cells and cell lines which exhibit downregulated expression of LZTS1^35,36^ like MCF-7. Cyclophosphamide (Cytoxan) is a prodrug that is activated when metabolized via 4-hydroxylation in the liver^34^. Although it is potent to breast cancer cell in vivo, it does not work before it is activated like an in vitro environment. Cisplatin operates well in 2D cell culture, nevertheless, it becomes ineffective when breast cancer cells acquire the cell-ECM contact, which is established in a 3-D context^37^.

**Figure 5:**
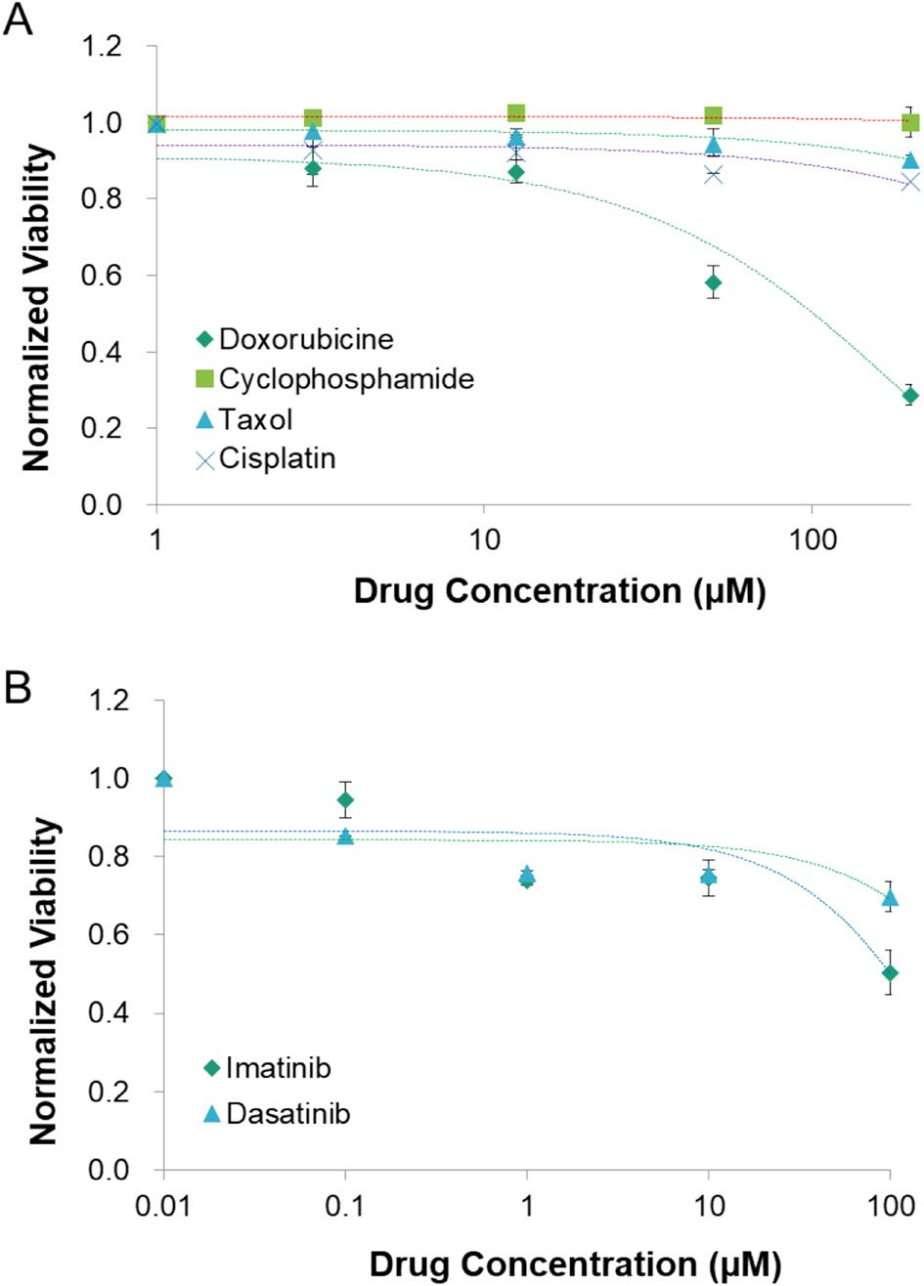
Efficacy test of the different drugs at various concentrations for adherent MCF-7 cells (**A**) and non-adherent K562 cells (**B**) using the device. The error bars represent the standard deviation for each concentration (n = 4).

Based on the drug efficacy test results presented in Fig. 5A, only doxorubicin exhibited potency against MCF-7 cells. The remaining three drugs displayed minor effectiveness, even at high concentrations. The differences in the cell response confirmed that, among the tested drugs, doxorubicin was the only effective agent against MCF-7 cells. In contrast, the other drugs were incompatible, suggesting that the device can discriminate potent drug and less effective drug although they were all chemo-drugs for breast cancer.

Besides adherent cells, drug efficacy could be assessed in the same manner for non-adherent cells. The leukemia cell line, K562, was tested against imatinib (Gleevec) and dasatinib (Sprycel). Although the K562 cells did not form a clustered structure owing to their natural characteristics, the ECM-like gel could trap the cells in the same manner. Moreover, the functional mechanism remained the same (Fig. S2B). The viability of K562 cells did not decrease when treated with 1–10 μM for both drugs (Fig. 5B). Though the viability decreased as drug concentration increased, it did not fall below 50%, even when the concentration of both drugs increased to 100 μM, which is above the IC_50_ of both drugs^38,39^. The reason for these differences might be that the ECM-like gel, which trapped the non-adherent K562 cells, acted as a barrier for drug diffusion.

### On-chip multi-drug efficacy test

**Figure 6:**
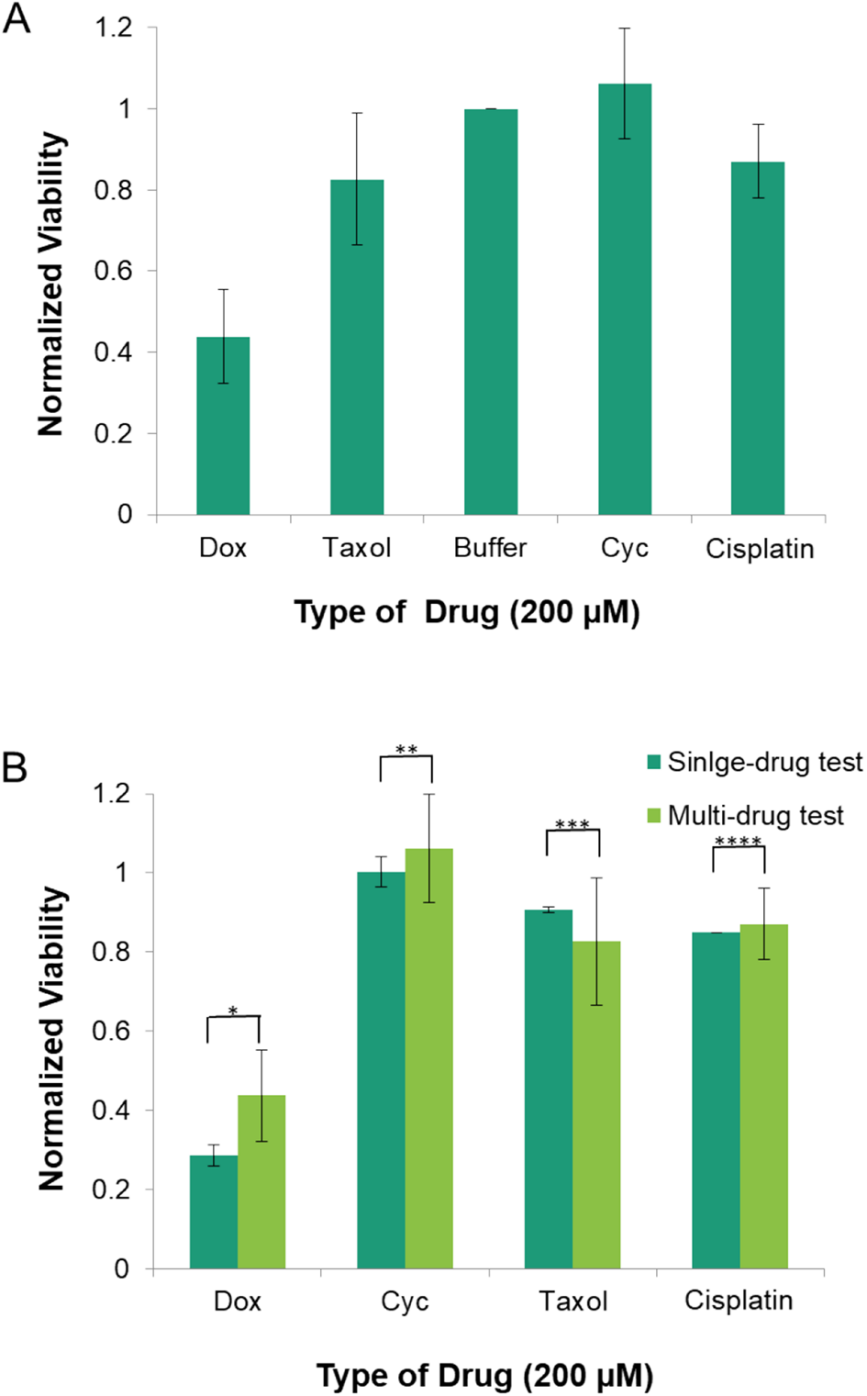
Simultaneous multi-drug efficiency test using the device. (Dox: doxorubicin; Cyc: cyclophosphamide; Taxol: paclitaxel; Cisplatin: cisplatin) (**A**) Normalized viability of cells located over each test lane loaded with the different drugs. (**B**) Comparison of the normalized viability for each drug according to the test format. The p-values for each drug were 0.07 (*, Dox), 0.17 (**, Cyc), 0.32 (***, Taxol), and 0.64 (****, Cis-Platin). The error bars represent the standard deviation for each drug (n = 4).

Next, a multi-drug efficacy test was performed using the four anticancer drugs, doxorubicin, paclitaxel, cyclophosphamide, and cisplatin. The concentration of each drug was fixed at 200 μM, and all drugs were delivered over the same time duration in the designated drug channel. As our platform consisted of five drug channels, the four drugs and one control were tested. When cell viability was examined in this experiment, the cells were found to be more susceptible to doxorubicin, followed by paclitaxel and cisplatin, whereas cyclophosphamide treatment was found to be ineffective (Fig. 6A). When the results of the single drug test were compared to those of the different concentrations, the ranking of the drugs and the cell viability values were not different (p-value of two-tailed *t*-test > 0.05) (Fig. 6B). Therefore, our platform could be used to quantitatively assess whether a drug is effective or ineffective as chemotherapy treatment for breast cancer.

### Potential use of real-time monitoring of cell viability

**Figure 7:**
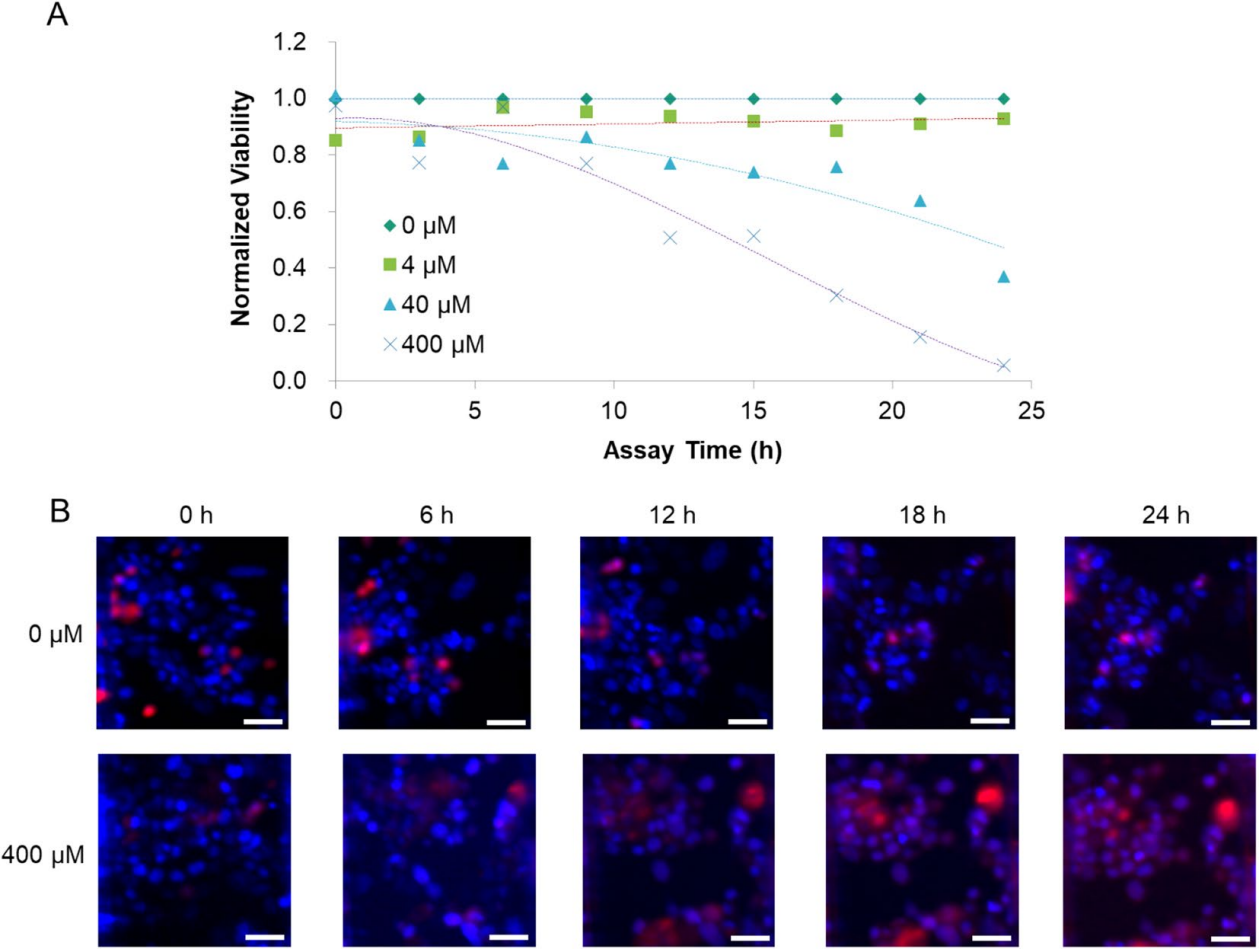
Real-time monitoring of cell viability using the device. (**A**) Time-dependent normalized viability of cells (MCF-7) in each test lane treated with different concentrations of drug (doxorubicin). (**B**) Stained images of cells located over the test lane with 0 and 400 μM doxorubicin at the indicated time intervals (Blue: Hoechst, Green: calcein-AM, Red: ethidium homodimer I) (Scale bar = 50 μm). Unmerged images are provided in supplemental information (Fig. S3B).

Real-time monitoring of drug efficacy may provide informative cytotoxicity kinetic data to aid in the classification of drugs according to their cytotoxic mode of action^40^. Considering the characteristics of our platform, real-time monitoring of drug efficacy could be conducted by loading the drug and staining reagent together in the drug channel, and then incubating the device in a microscope equipped with a stage-top incubator. Unlike in a prior experiment where cell viability was measured as the end-point, in this study, the reagent could not be used to stain live cells (calcein-AM) because of its spontaneous hydrolysis in the medium, which resulted in an increase in the background fluorescence intensity. To measure viability, we calculated the number of live cells indirectly by subtracting the number of dead cells stained with EthD-1 from the total number of cells stained with Hoechst. The viability and merged images of cells exposed to different concentrations of drugs at each time interval are presented in Fig. 7. These results demonstrated the typical dependency of drug efficacy at different concentrations over periods of time. Owing to the cytotoxicity of the staining reagent itself^41,42^, as shown by the non-normalized viability in Fig. S3A, the time duration of the assay in the previous experiment (48 h) could not be used. Thus, we conducted a real-time monitoring experiment over a shorter time duration (24 h) using a high drug concentration to compensate for the time limitations of the assay. Nevertheless, as shown in Fig. 7A, the decline in viability based on the dose was discriminative, enabling the determination of efficacy.

## Discussion

In this study, an open-chamber, multi-channel microfluidic device was developed to test different chemotherapy drugs in treatment regimens. Cells were attached to the membrane and covered with Matrigel in conjunction with a microchannel network, allowing localized drug delivery. Unlike other 3-D cell formations that use a cell–matrix solution in microfluidics, in our platform, a large part of the cells was bound to the membrane, whereas the remainder of the cells were bound to those cells. Therefore, at the end of the experiment, most cells were focused and imaged along the z-axis. As a result, we could easily obtain a microscopic image, similar to 2-D, in vitro cell culture tests. In addition, our platform could handle samples that were not fully dissociated, such as cell clusters, microtissues, and tissue slices.

Our numerical analysis suggested that the alternative placement of the drug and buffer channels with space enabled localized chemo-invasion in a cross-contamination-free manner. Although the patterns and values of the experimental results did not fully align with those of the simulation, as the latter could not represent all occurring phenomena such as the interaction between Hoechst and the ECM gel, they displayed sufficient concordance with the numerical analysis.

ECM-based 3-D culture is known to cause lower sensitivity to chemotherapeutic agents, which results in higher IC_50_ values than those obtained in 2-D monolayer-based drug tests^43–45^. This finding has been attributed to several factors, such as the decreased penetration of drugs, increased pro-survival signaling, and upregulation of genes conferring drug resistance, all of which are attributed to cell–cell and cell–matrix interactions^46,47^. The growth rate of breast cancer cells in a 3-D culture may also alter the inhibitory activity of a drug, especially as the cytotoxic effects may be weaker in cells with a slow doubling time than in those that proliferate rapidly^48^. Our ECM-like gel system, which could trap the cells, was expected to impose these cellular behaviors in a similar manner. This assumption was supported by the IC_50_ of doxorubicin for MCF-7 cells, which was comparable to the value obtained in a recent study using a 3-D context^33^. As our on-chip drug efficacy test results adequately reflected this biology, it was possible to avoid an overestimation of potency. The ability of the assay to use either suspended or adherent cell lines suggests that it can potentially be used in all cancer cell types.

Based on our findings, our platform can be used to rapidly assess drug potency, thereby enabling clinicians to determine an appropriate therapeutic regimen within ~ 2 days following tumor biopsy or resection. In addition, the potential use of real-time monitoring of cell viability was also demonstrated. Compared with commercial real-time monitoring platforms, which measure viability using an indirect assay^49^, the merits of our platform include the direct measurement of viability by staining cells at a single cell level and examining their morphology. Although there are some limitations, including the spontaneous cell death induced by the cytotoxicity of the staining reagent itself, the potential to perform real-time monitoring of drug efficacy—which will allow faster decision making—was successfully demonstrated.

In conclusion, we developed a permeable membrane-based microfluidic platform that enabled the efficient evaluation of the susceptibility of cancer cells to drugs. Our platform allowed multiplexed testing in a single chamber and required a small number of cells, which can be easily acquired from a biopsy. As demonstrated herein, our platform could successfully evaluate drug efficacy within 48 h or less if monitored in real-time. Therefore, our technology could enable evidence-based decision-making to support personalized cancer therapy. We believe that this approach will greatly promote the use of microfluidic technologies in precision medicine.

## Methods

### Device fabrication

The device was fabricated by photolithography and soft lithography. As the device consisted of several layers, two SU-8 molds were patterned to form the upper and bottom parts. First, SU-8 100 photoresist (MicroChem, USA) was spin-coated onto a Si wafer and patterned using photolithography. This mold was used to cast the upper part, which included the open cell chamber and inlet/outlet holes. A mixture of PDMS prepolymer (Sylgard 184) and curing agent (weight ratio, 10:1; Dow Corning, USA) was poured and cured onto the mold at 80 °C for 1 h in a dry oven and then peeled off from the mold. The open cell chamber and inlet/outlet holes were bored using a custom-made punch and a 2 mm biopsy punch (Ted Pella, USA), respectively.

The bottom part, which comprised a two-layer channel network and an open channel for reagent transfer, was cast on a mold made using multilayer photolithography. SU-8 100 photoresist was spin-coated onto a Si wafer at a thickness of 100 µm and exposed to UV under a photomask of the channel network. Thereafter, an additional 100 μm of SU-8 100 was spin-coated onto the exposed SU-8 layer and exposed to UV under a photomask of the open channel; both layers were developed simultaneously. A precise volume of a mixture of PDMS prepolymer and curing agent was poured onto the mold to adjust the height of the PDMS mixture to ensure that it did not exceed the height of the SU-8 mold to allow open channels. Thereafter, it was cured under the same conditions as the upper part and peeled off. The PDMS replica was bound to a 70 × 50 mm slide glass substrate using O_2_ plasma treatment.

A transparent track-etched membrane filter (ipCELLCULTURE, It4ip, Belgium) with a 0.45 μm pore was cut and treated with O_2_ plasma and a maintaining agent. The upper and bottom parts were also treated with O_2_ plasma and assembled with the membrane filter. Finally, the assembled device was sterilized using ethylene oxide and sealed until the initiation of the experiment.

### Numerical simulation of drug diffusion

The concentration profiles of drug diffusion from each drug-release channel within the platform during chemo-invasion were characterized using COMSOL Multiphysics (COMSOL Inc., Sweden) with the Transport of Diluted Species module. Owing to the symmetrical structure of the chamber and the time dependency of the diffusion, a 2-D transient analysis was conducted. The diffusion coefficient of Hoechst in the Matrigel and media was assumed to be 2.57 × 10^–11^ m^2^/s and 4.93 × 10^–10^ m^2^/s, respectively (Table S1). The concentrations of the molecules within the drug-release channel and buffer channel were set to 0–200 μM/mL and 0 μM/mL, respectively. The permeable membrane was set as a porous medium with 0.6% porosity and 12 μm thickness to match the dimensions of the filter used. As the sub-micron pores created a very high hydrodynamic resistance, convection through the membrane was negligible. Owing to the gel matrix filled in the cell chamber, convection within the chamber was also negligible.

### Cell preparation

Two cell lines, MCF-7 (human breast cancer; adherent) and K562 (human leukemia; non-adherent), were obtained from the ATCC. The cell lines were grown in RPMI-1640 (K562) or DMEM (MCF-7) supplemented with 10% FBS and 1% PS and incubated in a 5% CO_2_-humidified atmosphere at 37 °C. MCF-7 cells were harvested for use by trypsinization and then resuspended in phenol-free DMEM (FluoroBrite, #A18967-01, Gibco, USA) supplemented with 10% FBS and 1% PS. K562 cells were resuspended in phenol-free RPMI-1640 (#11835–030, Gibco) supplemented with 10% FBS and 1% PS. A phenol-free medium was used for optical transparency.

### Device operation for drug tests

Before cell loading, the cell chamber of the device was coated with a cell adhesive (Cell-Tak, #354240, Corning, USA) in accordance with the manufacturer’s protocol. After washing with DI water and drying, the prepared cells were pre-stained with 1 μM Hoechst 33,342 and the cell chamber of the device was loaded with 1 × 10^4^ cells/mL. The cells were incubated for 3 h to enable attachment. After removing 700 μL of the medium, 300 μL Matrigel (Phenol-free, Growth factor reduced, BD Biosciences, USA) was added to cover the cell layer. Thereafter, the device was incubated for 1 h to allow for Matrigel gelation.

The microfluidic channel was pre-coated with a 1% polyvinylpyrrolidone (PVP; in DI water) solution to form a hydrophilic surface. Doxorubicin (#D1515, Sigma, USA), paclitaxel (#S1150, Selleckchem, USA), and cyclophosphamide (#PHR 1404, Sigma, USA) were dissolved in phenol-free DMEM to obtain the desired drug concentrations. As many drugs are often dissolved in dimethyl sulfoxide (DMSO) before use, the final DMSO concentration was set to 1% for every sample, including the control. Imatinib (#SML1027, Sigma, USA) and dasatinib (#S1021, Selleckchem, USA) were dissolved in phenol-free RPMI, and the drug concentration was set to 1%. Each drug solution was injected into the designated channel and transferred to the cell chamber through a membrane. The culture medium for the sink buffer was introduced into the buffer channels using a syringe pump. Each experimental step was conducted on a clean bench, and the device was placed inside a culture dish (and covered with a lid) before placing it in incubator to prevent evaporation while the cultured cells were exposed to the drug for 48 h.

### Cell staining, imaging, and analysis

After drug-release, the channels were flushed with PBS. Thereafter, 40 μM EthD-1 and 20 μM calcein-AM solution (Live/Dead cell staining kit, Invitrogen, USA) were loaded into each channel for a 2 h incubation. The Live/Dead staining solution in the microchannel was flushed and washed with PBS before imaging.

Fluorescence images were acquired using a fluorescence microscope (IX81, Olympus, Japan) equipped with an automated stage (SCAN IM, Märzhäuser, Germany) and a cooled CCD camera (ORCA-R2, Hamamatsu, Japan). Images were stitched together to generate scanned images of the lane where the drug and buffer channels lay beneath or the full cell chamber, depending on the experiment. As the cells formed a 3-D structure, images were captured in double layers with 30 μm step-sized directional z-stacks.

Cell counting was either performed manually or with the analysis tool from the imaging software (CellSens Dimension, Olympus, Japan). Live cells were stained with Hoechst (nuclei) and calcein-AM (cytosol), whereas dead cells were stained with Hoechst and EthD-1 (nuclei). The region of interest (ROI) was set as each channel lane where the drugs and medium were released. Cell viability was calculated after the live and dead cells were counted.

### Real-time monitoring of drug efficacy

Real-time monitoring of drug efficacy was carried out using a microscope stage-top incubator (Chamlide, Live Cell Instrument, South Korea) equipped with a temperature controller, gas mixer, humidifier, and incubator chamber. Briefly, doxorubicin (400 μM, 40 μM, 4 μM, and 0 μM) with 20 μM EthD-1 was introduced through each drug channel. Images were captured every 3 h, and the cell viability was monitored over time.

## Supplementary information

Supplementary Information 1.
