# Supplementary material for: Application of an open-chamber multi-channel microfluidic device to test chemotherapy drugs
Source: Sci Rep. 2020 Nov 23;10:20343. doi: 10.1038/s41598-020-77324-3 (PMC7683738; doi:10.1038/s41598-020-77324-3)
Supplement: Supplementary file 1 — Supplementary Information 1. [file 41598_2020_77324_MOESM1_ESM.docx]

**Supplementary Information**

# Title:

**Application of an open-chamber multi-channel microfluidic device to test chemotherapy drugs**

Hui-Sung Moon^1†*^, Chang Eun Yoo^1†**^, Sangmin Kim^2^, Jeong Eon Lee^3,4^, Woong-Yang Park^1,4,5***^

^1^Samsung Genome Institute, Samsung Medical Center, Seoul 06351, South Korea

^2^Department of Breast Cancer Center, Samsung Medical Center, Seoul 06351, South Korea

^3^Department of Surgery, Samsung Medical Center, Seoul 06351, South Korea

^4^Department of Health Sciences and Technology, SAIHST, Sungkyunkwan University, Seoul 06351, South Korea

^5^Department of Molecular Cell Biology, Sungkyunkwan University School of Medicine, Suwon 16419, South Korea

^†^These authors contributed equally to this work

* Corresponding author. hs.moon@samsung.com

** Corresponding author. changeun.yoo@samsung.com

*** Corresponding author. woongyang.park@samsung.com

**Stokes-Einstein relationship: *D* = *kT*/6πη*R***

|  | Matrigel | Culture medium |
| --- | --- | --- |
| R, Stokes radius (Hoechst) (m) | 5.90 × 10^-10^ | |
| k, Boltzmann constant (J/K) | 1.38 × 10^-23^ | |
| T, Temperature (K) | 310.15 | |
| η, Dynamic viscosity (Ns/m^2^) | 1.50 × 10^-2^ ^50^ | 7.80 × 10^-4^ ^51^ |
| **D, Diffusion coefficient (m^2^/s)** | 2.57 × 10^-11^ | 4.93 × 10^-10^ |

Table S1. Summary of parameters used to calculate diffusion coefficients. Stokes radius was assumed from calculations based on molecular weight.


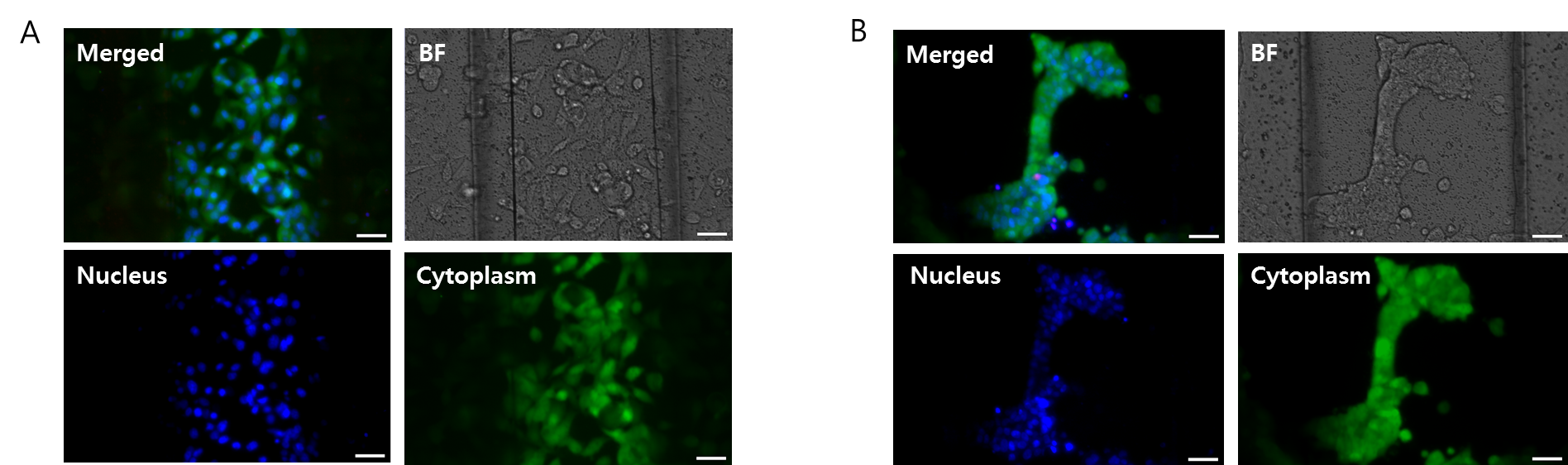


Figure S1. On-device cell culture image. (A) 2D cell culture without ECM matrix (B) 3D cell culture with ECM matrix. Hoechst stain was used to stain the nucleus, and calcein-AM was used to stain the cytoplasm. (scale bar = 50 μm)


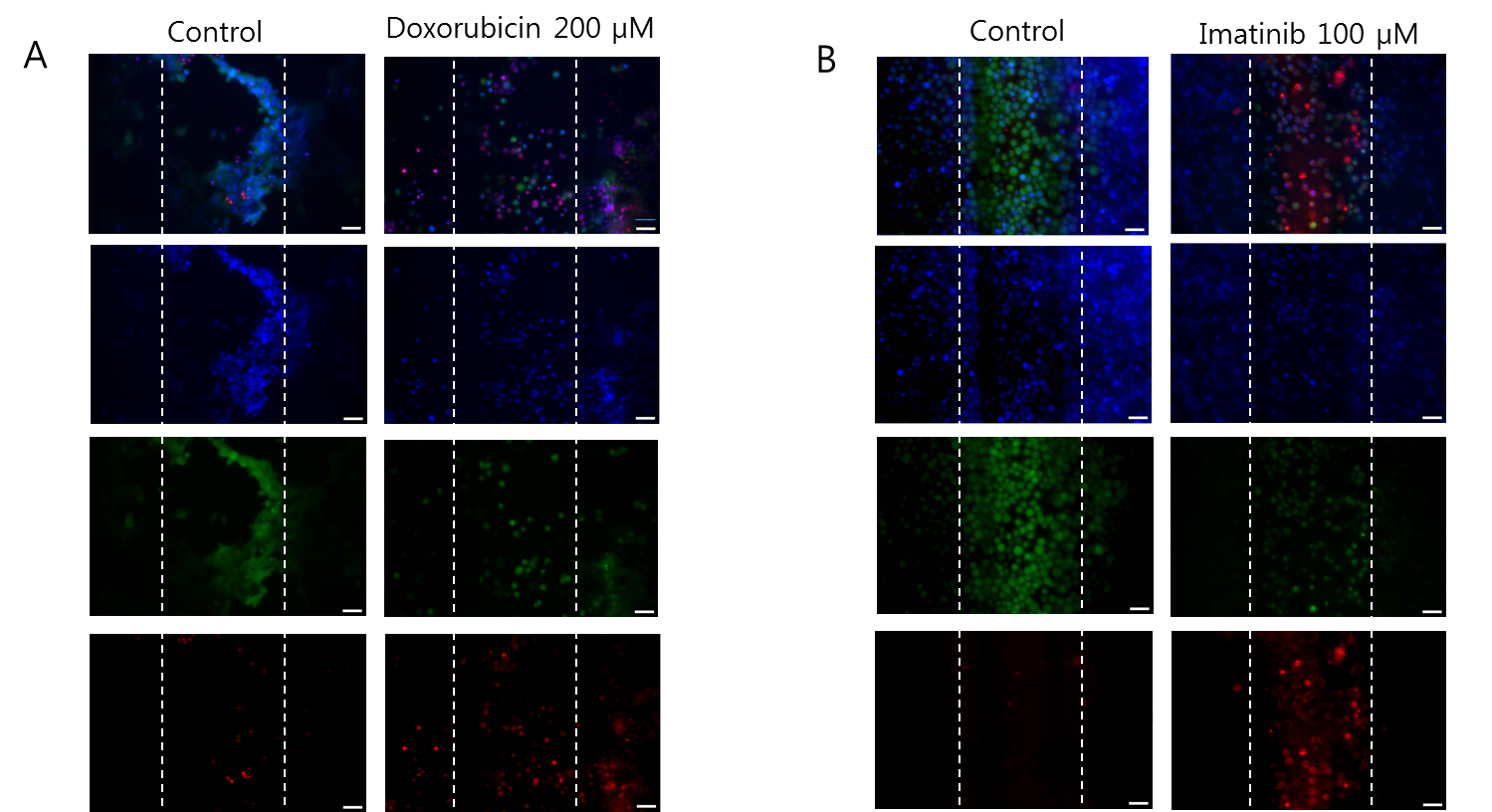


Figure S2. Stained image of (A) adherent cells (MCF-7) and (B) non-adherent cells (K562) in the test and control lanes. (Blue: Hoechst, Green: calcein-AM, Red: ethidium homodimer I). The dashed lines in each image represent virtual boundaries that indicate the lanes with drugs (test lanes) or buffer (sink lanes). (scale bar = 50 μm)


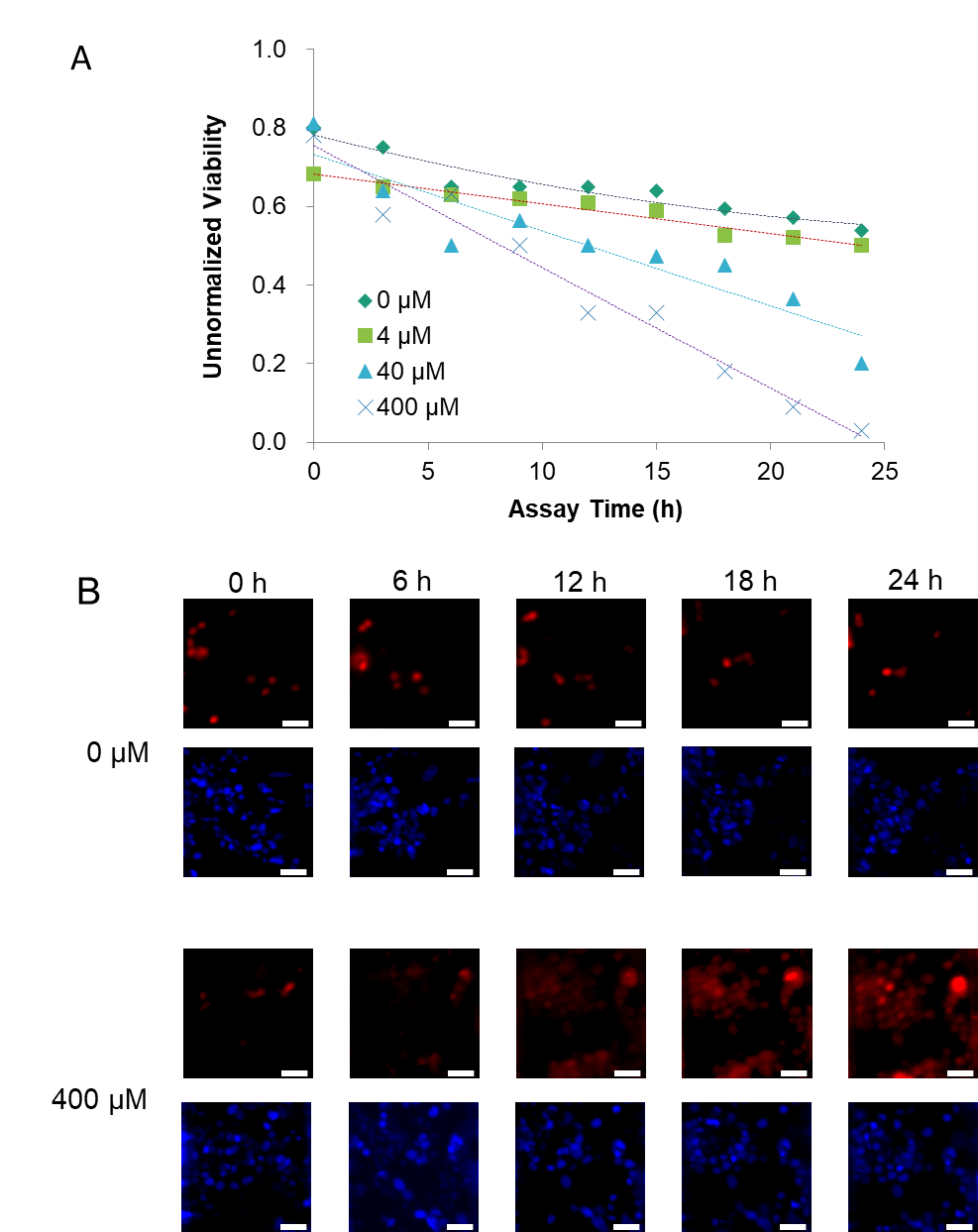


Figure S3. Real-time monitoring of cell viability using the device. (A) Time-dependent unnormalized viability of cells (MCF-7) in each test lane with different concentrations of drug (doxorubicin). (B) Stained images of cells located over the test lanes with 0 and 400 μM doxorubicin at the indicated time intervals (Blue: Hoechst, Green: calcein-AM, Red: ethidium homodimer I, unmerged). (scale bar = 50 μm).

# Reference

50 Sawonik, M. A. *A microfluidic platform to quantify spatio-temporal diffusion of chemo-gradients within 3d scaffolds: Applications in axonal biology*, Cleveland State University, (2013).

51 Wang, C., Lu, H. & Schwartz, M. A. A novel in vitro flow system for changing flow direction on endothelial cells. *Journal of biomechanics* **45**, 1212-1218 (2012).
